# AcrNET: predicting anti-CRISPR with deep learning

**DOI:** 10.1093/bioinformatics/btad259

**Published:** 2023-04-21

**Authors:** Yunxiang Li, Yumeng Wei, Sheng Xu, Qingxiong Tan, Licheng Zong, Jiuming Wang, Yixuan Wang, Jiayang Chen, Liang Hong, Yu Li

**Affiliations:** Department of Computer Science and Engineering, The Chinese University of Hong Kong, Sha Tin, Hong Kong SAR 999077, China; Department of Computer Science and Engineering, The Chinese University of Hong Kong, Sha Tin, Hong Kong SAR 999077, China; Department of Computer Science and Engineering, The Chinese University of Hong Kong, Sha Tin, Hong Kong SAR 999077, China; Department of Computer Science and Engineering, The Chinese University of Hong Kong, Sha Tin, Hong Kong SAR 999077, China; Department of Computer Science and Engineering, The Chinese University of Hong Kong, Sha Tin, Hong Kong SAR 999077, China; Department of Computer Science and Engineering, The Chinese University of Hong Kong, Sha Tin, Hong Kong SAR 999077, China; Department of Computer Science and Engineering, The Chinese University of Hong Kong, Sha Tin, Hong Kong SAR 999077, China; Department of Computer Science and Engineering, The Chinese University of Hong Kong, Sha Tin, Hong Kong SAR 999077, China; Department of Computer Science and Engineering, The Chinese University of Hong Kong, Sha Tin, Hong Kong SAR 999077, China; Department of Computer Science and Engineering, The Chinese University of Hong Kong, Sha Tin, Hong Kong SAR 999077, China

## Abstract

**Motivation:**

As an important group of proteins discovered in phages, anti-CRISPR inhibits the activity of the immune system of bacteria (i.e. CRISPR-Cas), offering promise for gene editing and phage therapy. However, the prediction and discovery of anti-CRISPR are challenging due to their high variability and fast evolution. Existing biological studies rely on known CRISPR and anti-CRISPR pairs, which may not be practical considering the huge number. Computational methods struggle with prediction performance. To address these issues, we propose a novel deep neural network for anti-CRISPR analysis (AcrNET), which achieves significant performance.

**Results:**

On both the cross-fold and cross-dataset validation, our method outperforms the state-of-the-art methods. Notably, AcrNET improves the prediction performance by at least 15% regarding the F1 score for the cross-dataset test problem comparing with state-of-art Deep Learning method. Moreover, AcrNET is the first computational method to predict the detailed anti-CRISPR classes, which may help illustrate the anti-CRISPR mechanism. Taking advantage of a Transformer protein language model ESM-1b, which was pre-trained on 250 million protein sequences, AcrNET overcomes the data scarcity problem. Extensive experiments and analysis suggest that the Transformer model feature, evolutionary feature, and local structure feature complement each other, which indicates the critical properties of anti-CRISPR proteins. AlphaFold prediction, further motif analysis, and docking experiments further demonstrate that AcrNET can capture the evolutionarily conserved pattern and the interaction between anti-CRISPR and the target implicitly.

**Availability and implementation:**

Web server: https://proj.cse.cuhk.edu.hk/aihlab/AcrNET/. Training code and pre-trained model are available at.

## 1 Introduction

Anti-CRISPR is an important group of proteins discovered in phages for fighting against the immune system of certain bacteria. To resist the invasion of phages, bacteria have evolved different types of defense mechanisms, including the important and adaptive immune system CRISPR-Cas. Correspondingly, phages evolved inhibitor proteins anti-CRISPRs (Acrs) to fight with the CRISPR-Cas system. Because of the strong ability to adaptively detect, destroy, and modify DNA sequences, CRISPR-Cas has become a popular gene-editing tool. Since there could be a dedicated Acr available for each CRISPR-Cas system (Stanley and Maxwell 2018), performing accurate Acr predictions to find new Acrs is becoming increasingly important for many real-world applications, such as reducing off-target accidents in gene editing, measurement of gene drive, and phage therapy (Pawluk et al. 2018; Stanley and Maxwell 2018; Marino et al. 2020).

For the Acr prediction task, i.e. predict whether or not the given protein sequence is an Acr, many biological and bioinformatic approaches have been adopted to predict and discover new Acrs. Based on a plasmid-based functional assay, the activity of prophage, which integrated phage genome and bacterial genome, was observed and evaluated, leading to the discovery of the first Acr that enabled phage to replicate successfully under CRISPR-Cas attack (Bondy-Denomy et al. 2013). By utilizing the BLAST search strategy on anti-CRISPR-associated (Aca) genes, which is an important characteristic of certain Acr genes, Pawluk et al. (2016) developed a bioinformatic approach to find additional Acr proteins in phages and related diverse mobile genetic data in bacteria. Motivated by the idea that “self-targeting” prophage contains both a DNA target and CRISPR spacer as the CRISPR-Cas system is inactivated, Rauch et al. (2017) conducted a study to search bacterial genomes for the co-existence of the spacer and its target, discovering new Acrs in phages with this self-targeting phenomenon. A phage-oriented approach led to an Acr only for abolished immunity which is unrelated to previous Acrs (Hynes et al. 2017). However, these methods depend on expensive and time-consuming experimentally generated data. Furthermore, they rely heavily on the homologs of Acrs and their functional characteristics, which is not practical for the rapid emergence of a large number of new types of proteins.

Several machine learning (ML) methods were introduced to predict Acrs and accelerate biological discoveries. An ensemble learning-based Acrs prediction tool, PaCRISPR (Wang et al. 2020), was developed to apply the support vector machine model on evolutionary features, which were extracted from Position-Specific Scoring Matrix (PSSM), including PSSM-composition (Zou et al. 2013), DPC-PSSM (Liu et al. 2010), PSSM-AC (Dong et al. 2009), and RPSSM (Ding et al. 2014). A new Acrs prediction model named AcRanker (Eitzinger et al. 2020) based on XGBoost ranking was built to deal with the mixture of different sequence features, including grouped dimer, amino acid composition, and trimer frequency counts (Eitzinger et al. 2020). A server, AcrFinder was built to pre-screen genomic data for Acr candidates by combining three well-accepted ideas: guilt-by-association, homology search, and CRISPR-Cas self-targeting spacers (Yi et al. 2020). An ML approach consisting of a random forest with 1000 trees was built to predict comprehensive Acrs, which showed strong forecasting capability for the unseen test set (Gussow et al. 2020). Also built upon the random forest, AcrDetector utilized merely six features to identify Acrs from the whole genome scale but still maintained the prediction precision (Dong et al. 2020). As described in the AcrHub paper, it incorporates state-of-the-art predictors and three functional analysis modules, including homology network analysis, phylogenetic analysis, and similarity analysis (Wang et al. 2021). These models are relatively flexible compared with the traditional bioinformatic approaches. However, these methods usually rely on traditional ML techniques, which utilize simple linear models or shallow non-linear models, limiting their modeling capacity making it difficult to promote the development of Acr-based precise treatment. DeepAcr (Wandera et al. 2022) applies deep learning models, where they only use simple protein features, lacking the ability to solve the data scarcity problem.

Considering the scarce database, Acrs’ quick evolution, and under-explored Arc features, we propose a novel deep learning approach (AcrNET) for the effective and accurate prediction and classification of Acr proteins to facilitate new Acr discovery from large-scale protein databases. The biggest challenge of developing deep learning methods to predict Acrs is the lack of data and none of previous works took this issue into account. For example, we only have 1094 non-redundant Acr sequences in our dataset, which is usually insufficient for an effective deep learning model. To deal with the data scarcity issue, we adopt a pre-trained large-scale Transformer protein language model that was developed by Rives et al. (2021), making it possible to leverage highly informative protein representations The pre-trained model extracts internal statistical properties to effectively promote the prediction performance for structure, function, and other tasks (Suzek et al. 2015; Rives et al. 2021). This learning model explored 250 million protein sequences, significantly broadening our database and helping achieve better prediction performance. Meanwhile, this model is computationally efficient, which can even meet the time requirement used for tremendous protein sequences. Protein language models have been applied in many downstream tasks and led to improvement, including protein–protein interaction (Sledzieski et al. 2021) and residue–residue contact prediction (Rao et al. 2021). Furthermore, to fully utilize valuable information of protein data to promote the performance, we combine the protein language model feature with various other features, including Acr sequence information, Acr protein structure information, relative solvent accessibility, and four evolutionary features from PSSM (PSSM-composition, DPC-PSSM, PSSM-AC, and RPSSM), which are proved helpful for Acrs prediction (Eitzinger et al. 2020; Wang et al. 2020). We also provide more detailed and informative hierarchical classification results compared with the current classification scheme in the anti-CRISPR system, which broadly divides proteins into two categories according to whether a protein is Acr or not (Koonin et al. 2017). At the first level, similar to the current anti-CRISPR system, we predict whether a protein is an Acr. Then, at the second level, if it is an Acr protein, we further provide which class of Acrs the protein belongs to, which can bridge the large-scale protein database and Acrs to accelerate the discovery and verification of Acrs. Down to the details of the whether a protein is an Acr prediction (prediction task) and Acr type classification procedure (classification task), we develop the model based on convolutional neural networks (CNNs) and fully connected networks (FCNs).

Our contributions are as follows.

- Based on the datasets from anti-CRISPRdb (Dong et al. 2018) and PaCRISPR (Wang et al. 2020), we propose a deep learning method, AcrNET, for anti-CRISPR prediction, which outperforms the previous state-of-the-art methods by at least 15% regarding the F1 score on cross-dataset validation.
- We develop the first computational method that can predict the detailed anti-CRISPR classes, which may help illustrate the anti-CRISPR mechanism.
- We are the first ones in this field to resolve the data scarcity issue by transferring the knowledge from a large-scale protein language model trained on 250 million protein sequences.
- We perform extensive experiments and analysis to investigate the relation among the Transformer model feature, evolutionary feature, and local structure feature, which suggests that they complement each other, indicating the critical properties of anti-CRISPR proteins.
- Combining AcrNET with AlphaFold and motif detection methods, we propose a computational pipeline to understand the model prediction basis better and validate our prediction computationally.

## 2 Method

### 2.1 Overview of AcrNET

**Figure 1.**
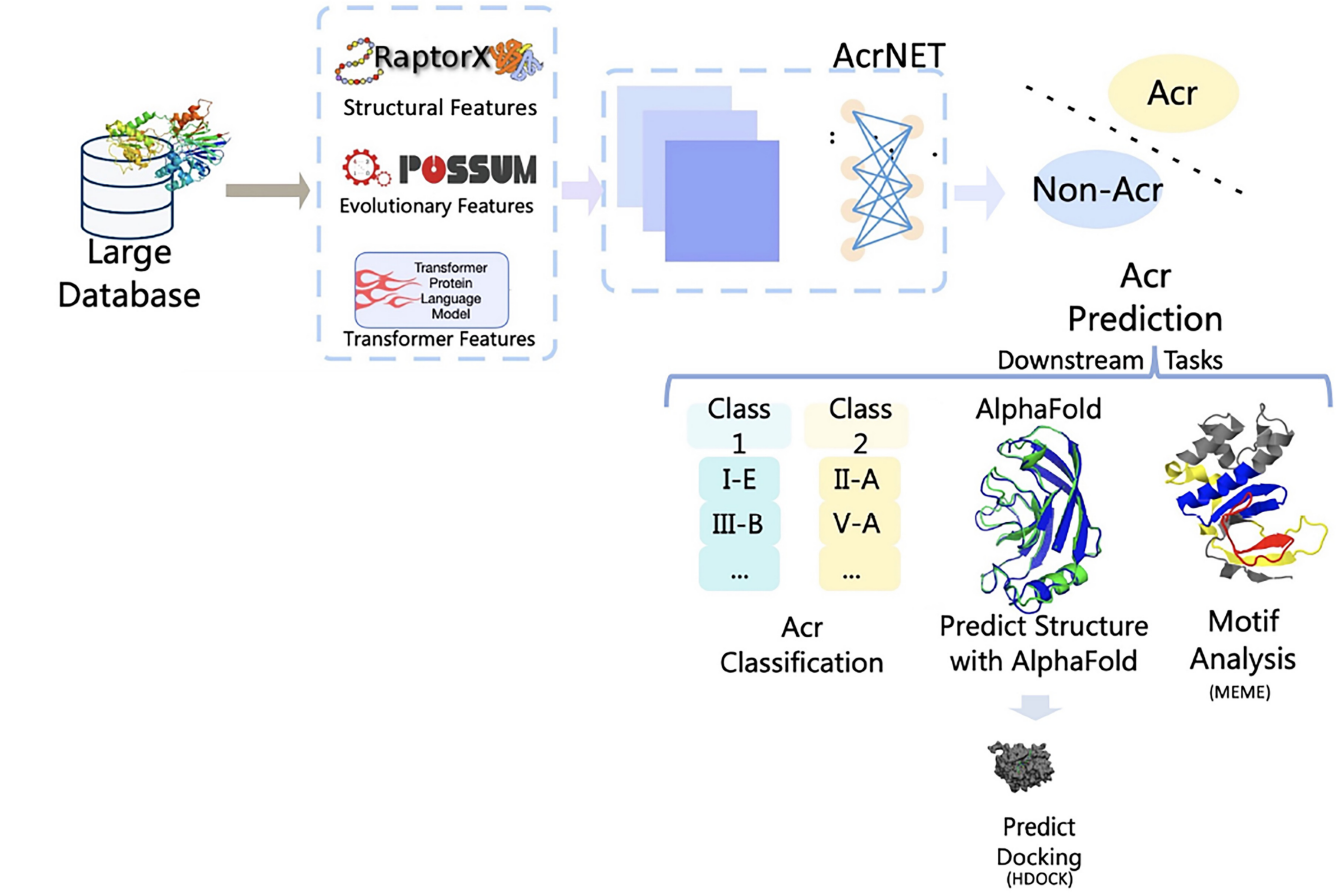
The AcrNET pipeline. The process contains three steps. First, we compile the large protein sequence database to obtain secondary structure, relative solvent accessibility, evolutionary features, and Transformer features. Second, based on the features, the AcrNET model predicts the Acr proteins. Finally, with the predicted likelihood of the protein being an Acr protein, we can perform multiple downstream tasks, including predicting classes of Acrs, doing biological validation experiments, predicting the Acr structure with AlphaFold, investigating the interaction between Acr and the target with protein–protein docking, and motif analysis.

AcrNET contains three basic parts, as in Fig. 1. First, a large collection of protein sequences are compiled into the pipeline to extract protein features, including secondary structure, relative solvent accessibility, four evolutionary features, and Transformer features. Furthermore, by introducing the Transformer learning module, we tackle the scarcity issue of Acrs database with a large-scale pre-training database. Second, the learned features are injected into AcrNET, which jointly models these features using two deep learning modules, CNN and FCN, in an end-to-end trainable deep architecture. To this end, the model provides the confidence score of the likelihood that the protein is an Acr protein. Finally, based on the predicted likelihood, we can perform some downstream tasks. For example, positive Acr candidates are further inputted into AcrNET to perform the second-level prediction, predicting which sub-category the Acr belongs to. This sub-category prediction helps narrow down the candidate list, assisting biologists in carrying out biological experiments. We also conduct motif analysis to illustrate the implicit features learned by AcrNET. Additionally, we show the interaction details between the Acr protein and the target by predicting structure with AlphaFold and conducting protein–protein docking.

### 2.2 Extracting evolutionary features and structure features for comprehensive protein representation

Protein features necessary for Acr prediction are not well-defined. Thus, we focused on evolutionary and sequence features that have proven useful in earlier studies (Wang et al. 2020, 2021). We also incorporated the secondary structure, which is applied in protein function prediction (Li et al. 2018; Zou et al. 2018) and known to be related to protein–protein interaction (Jedhe and Arora 2021). First, we use one-hot encoding to encode the protein sequence, which contains original amino acid information, reflecting vital sequential patterns of Acr proteins. Second, we extract PSSM features to introduce evolutionary information of proteins. The PSSM score is an L×20 matrix summarizing the homologs similar to a given protein sequence in the specified database. Each score in the matrix reflects the conservation of the corresponding amino acid at this position, where large scores imply high conservation. From the PSSM matrix, we calculate four evolutionary features, PSSM-composition, DPC-PSSM, PSSM-AC, and RPSSM, which are fixed-size matrices. They deal with PSSM varying lengths, encode relation between two elements, and local sequence order effect (Wang et al. 2020). Third, we extract secondary structure information with traditional three classes (Pauling et al. 1951) and eight classes extended by Kabsch and Sander (1983), and convert them with one-hot encoding. We also consider solvent accessibility which presents the local exposure of a protein. We label them with three states and apply one-hot encoding. The last type of feature that we consider is Transformer feature, which helps consider the biological structure and function of protein data. During the process of learning such features, we can simultaneously handle the scarcity issue of Acrs database. We discuss it as a separate section in the following paragraph.

### 2.3 Introducing Transformer learning to tackle data scarcity

The proposed deep learning model has a large number of trainable parameters, i.e. 2 523 128 parameters in our model, which enables the model to have a very strong modeling capacity. However, it may suffer from the overfitting problem for data scarcity issue, especially for Acr prediction, where we only have around 1000 sequences. Thus, we adopt the Transformer learning algorithm to learn more informative representations (Rives et al. 2021), significantly broadening the training database. Unsupervised method has been introduced with the explosive growth of protein sequence data, which can capture statistical regularities (Hinton et al. 2006). This module learns the sequential information of the protein data by predicting the contents of the masked parts, a self-supervised learning approach, therefore guiding it to learn useful structure information from the sequential data to provide effective representations. Furthermore, because when we train the language model, we do not need the human-labeled data, we can train the model with as many protein sequences as possible. We adopt a protein language model trained over 250 million protein sequences from UniProt. The authors of Evolutionary Scale Modeling (ESM)-1b explored datasets with up to 250 million sequences of the UniParc database (Bairoch et al. 2007), which has 86 billion amino acids. The dataset they used owns comparable size to large text datasets that are being used to train high-capacity neural network architectures on natural (Devlin et al. 2019; Radford et al. 2019). They trained ESM-1b on the high-diversity sparse dataset (UR50/S). With such a huge amount of unsupervised training data, the model can implicitly learn the data distribution, evolutionary information, and protein sequential structure, leading to an effective protein representation. In addition, compared with the time and memory-consuming PSI-BLAST, POSSUM (Wang et al. 2017), and RaptorX (Källberg et al. 2012), the ESM-1b module (Yu et al. 2021, Chen et al. 2021) is able to generate Transformer features much more quickly and more simply.

### 2.4 Input data

We collected the anti-CRISPR data and the non-anti-CRISPR data from anti-CRISPRdb (Dong et al. 2018) and PaCRISPR (Wang et al. 2020), respectively. For positive anti-CRISPR data, anti-CRISPRdb contains ˃3000 experimentally characterized Acrs. We apply CD-HIT with 95% identity threshold to remove the redundant sequences and obtain 1094 non-redundant positive sequences. Non-Acr data in PACRISPR contains 1162 negative non-Acr sequences from Acr-containing phages or bacterial mobile genetic elements. The sizes of these samples range from 50 to 350. And the sequence similarity is <40%. Combining the positive and negative dataset, we obtain our complete dataset, which is the one for 5-fold experiments.

Furthermore, another cross-dataset test is conducted to evaluate the generalization ability of the proposed model for the Acr prediction, which is to evaluate a model’s performance on a test dataset that has different distribution from the training dataset. While in 5-fold cross-validation test, training and testing dataset are from the same distribution. To test the generalization ability of the model with cross-dataset test, we construct training and testing data with small similarities. For positive data, we separate the 1094 non-redundant positive samples in three different ways, we call each way as one separation. For separation 1, we selected I-F, II-C, and I-D types as testing samples, and the rest Acrs as training samples. For separation 2, we selected types I-F, I-E, V-A, I-C, VI-A, VI-B, III-I, III-B, and I-B as testing data. For separation 3, I-D, II-C, I-E, V-A, I-C, VI-A, VI-B, III-I, III-B, and I-B types are testing data. The specific arrangements are illustrated in Table 1. For negative dataset, the dataset has already been separated for cross-dataset test. The training dataset contains 902 sequences and testing data contains 260 sequences. In both positive and negative data, the similarity between the training samples and the testing samples is ˂40%.

**Table 1.** Statistics summary of the dataset used in our experiments.

	Cross-dataset training	Cross-dataset testing
Positive (separation 1)	884	210 (From type I-F, II-C, and I-D in anti-CRISPRdb)
Positive (separation 2)	904	190 (From type I-F, I-E, V-A, I-C, VI-A, VI-B, III-I, III-B, and I-B in anti-CRISPRdb)
Positive (separation 3)	962	132 (From type I-D, II-C, I-E, V-A, I-C, VI-A, VI-B, III-I, III-B, and I-B in anti-CRISPRdb)
Negative	902	260

We combine anti-CRISPRdb dataset and PaCRISPR dataset, and remove the sequence redundancy.

For downstream Acr type classification task, we use the 1094 non-redundant positive sequences. The 12 Acr types and the corresponding number of collections of each kind are shown in Table 2. In our design, we re-group these 12 types into five classes. Specifically, the samples in the first four types, namely II-A, I-F, I-D, and II-C, are maintained, while the rest eight types with very few samples are grouped into the “fifth type.” Therefore, as described in the Results section, the Acrs classification problem is a five-class classification task. The detailed organization of our dataset is shown in Supplementary Appendix Section 1.

**Table 2.** Statistical summary of the Acr classes in the non-redundant anti-CRISPRdb dataset.

Acr type	II-A	I-F	I-D	II-C	I-E	V-A	I-C	VI-A	VI-B	III-I	III-B	I-B
Collection	828	134	46	30	19	15	8	7	7	1	1	1

We keep the largest four classes and group the rest eight smaller classes as class 5.

### 2.5 Architecture for complex feature learning and Acr prediction and classification

**Figure 2.**
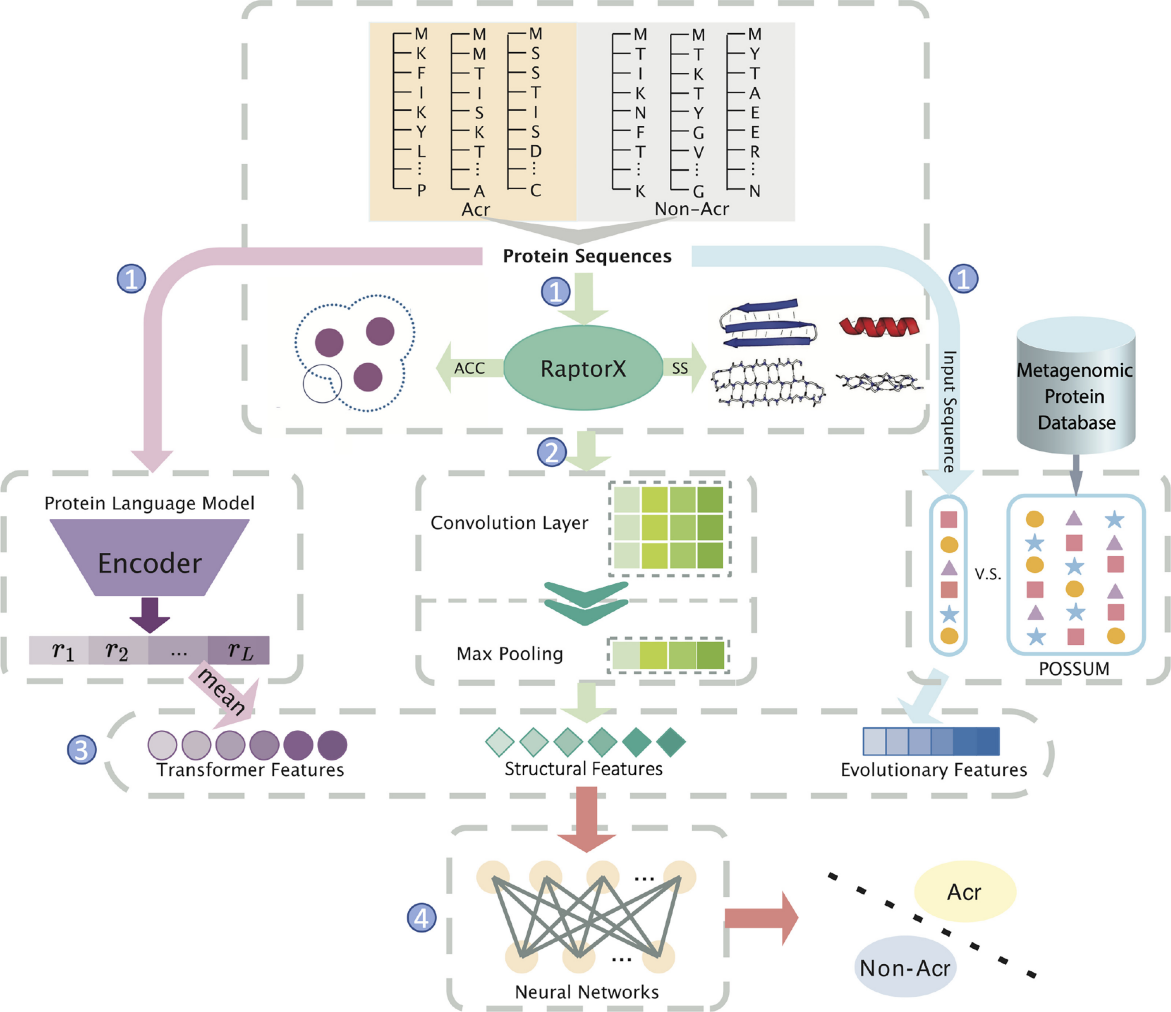
The architecture of the proposed AcrNET model. We first obtain the relative solvent accessibility, secondary structure, evolutionary features, and Transformer features with RaptorX, POSSUM, and a pre-trained protein language model. We input relative solvent accessibility, secondary structure into CNN, then concatenate the result with evolutionary features, and Transformer features and feed into FCN to obtain the predicted likelihood of the protein being an Acr protein. The numbers in the graph indicate the steps. Purple, green, and blue color imply processes of Transformer features, structural features, and evolutionary features, respectively.

Since original information from proteins is complex, we leverage CNNs and FCNs to learn useful features, designed in the end-to-end trainable way as illustrated in Fig. 2. First, we obtain structure feature, and solvent accessibility from RaptorX, Transformer features from ESM-1b and evolutionary features from POSSUM. Then, the one-hot matrices of sequence, structure feature, and solvent accessibility are concatenated together as the inputs of the CNN module. The convolutional layers learn deep features by extracting important local information from the one-hot matrices. After this, the max-pooling layer is attached along the sequential direction to calculate the largest value in each feature map. At the third step, the features learned by the CNN module are further combined with the evolutionary features and Transformer features from ESM-1b, which are then jointly modeled by the FCN module. Lastly, the Acr prediction is a binary classification task, which makes an estimation about whether a certain protein sequence is an Acr. Therefore, the final outputs are produced by fully connected layers with a two-unit output.

Considering biologists may also be interested in Acr types, if a protein is predicted to be an Acr, we will further estimate which kind of Acr this protein belongs to and provide more detailed information for the downstream biological experimental verification. Due to the limited number of Acr samples, we separate this task from Acr prediction to avoid the imbalanced data issue and defined it as the Acr classification task, predicting the specific category of the Acr. This classification task utilizes a similar structure to the Acr prediction task with slight modification, namely changing the dimension of the final output from two to five classes. In addition, we remove the non-anti-CRISPRs samples from the whole samples and only analyze samples predicted to be Acr proteins. We use cross-entropy as the loss function and Adam as the optimizer to train the model. Moreover, to increase the flexibility of the model for real-world applications, we make the model accept the input with variant features, which allows users to select the input features according to their needs.

### 2.6 Anti-CRISPR prediction performance

In this section, we discuss the performance of our model on predicting whether a certain protein sequence is an Acr or not. To sufficiently compare the prediction performance of our proposed method and existing models, we perform 5-fold cross-validation test and an additional cross-dataset test to evaluate their generalization ability. To exclude the influence of random seeds, we randomly generate seeds ten times for the initialization of model parameters and the split of samples of training and test groups. The final result of each model is their average of 10 times prediction results. Five evaluation metrics, namely accuracy (ACC), precision, recall, F1-score, and Matthews correlation coefficient (MCC), are utilized to evaluate the prediction results. We also report true positive (TP), false negative (FN), false positive (FP), TP, and specificity for ambiguous performance for further analysis. Since other methods require additional information other than the sequence alone, such as gene location on chromosome (Dong et al. 2020), we compare AcrNET with three recently proposed methods with the same dataset we collected, namely PaCRISPR (Wang et al. 2020), AcRanker (Eitzinger et al. 2020), and DeepAcr (Wandera et al. 2022). All of them have shown strong Acr predicting capacities and efficiency.

**Figure 3.**
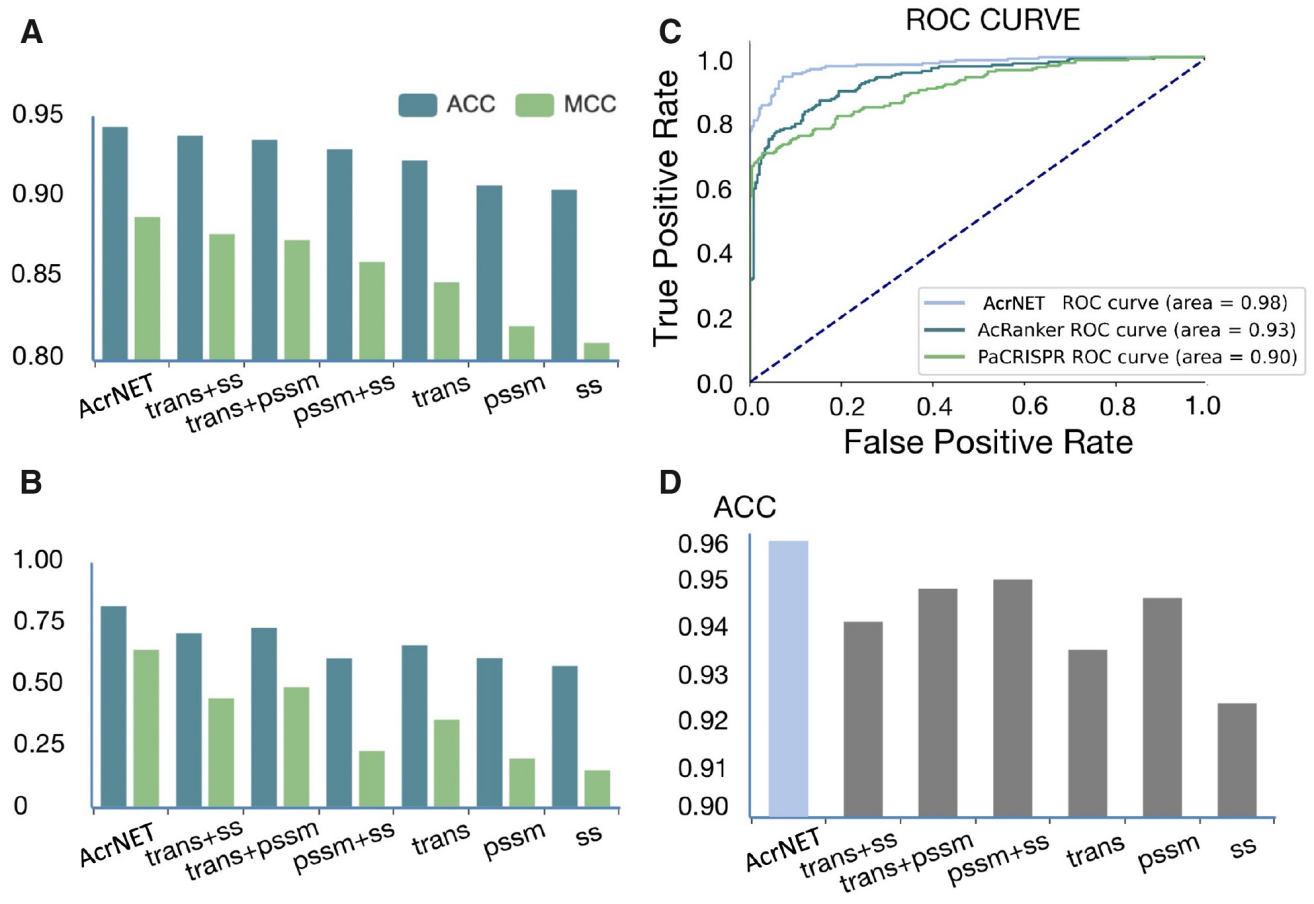
Feature influence on the prediction results. “ss” represents sequence encoding, 3-class secondary structure, 8-class secondary structure, and solvent accessibility; “trans” indicates Transformer features; “pssm” denotes 4 evolutionary features extracted from PSSM. Results in the figure are averaged over 10 different random seeds in our experiments. (A) Contribution evaluation of different features on five-fold cross-validation test. (B) Contribution evaluation of different features on cross-dataset test. (C) Performance comparison on Acr prediction regarding ROC curves of AcrNET, AcRanker, and PaCRISPR. (D) Contribution evaluation of different features for the detailed classification task.

The results of 5-fold cross-validation test and the cross-dataset test on different separations are demonstrated in Table 3, Table 4, and Supplementary Appendix Section 3, respectively, which clearly indicates that our proposed method outperforms PaCRISPR, AcRanker, and DeepAcr. Some important discoveries can be observed from these results. First, AcrNET substantially outperforms other methods in both tests, especially in the cross-dataset test. With respect to TP and TN, AcrNET with only Transformer features and the complete AcrNET are better on both TP and TN. The *P*-values for AcrNET performing better than PaCRISPR, AcRanker, and DeepAcr are all <0.0001. In the 5-fold cross-validation test, the F1 score is promoted by around 10%. The ROC curves also suggest that AcrNET is more robust than the other methods (Fig. 3C). Significantly, AcrNET achieves at least 15% improvement regarding F1 score in the cross-dataset test, where training and testing data have different distributions. We consider F1 score because it combines precision and recall and provides a more informative measure of a model’s performance than accuracy alone. The results demonstrate that AcrNET is more suitable for the real Acr prediction task. We assume that AcrNET method can automatically learn more useful knowledge from the protein data, including the important structure information, which enables AcrNET to outperform other models. Furthermore, the Transformer model that we adopt to our model contains common properties of protein sequences from an extensive database, which effectively improve the generalization ability for unseen sequences. Especially for cross-dataset test, pre-trained Transformer model may extract features from huge database which are rare in the small Acr dataset and difficult for normal ML or DL methods to learn. Besides, since the features are already provided, it reduces the chances that the deep model overfit on features from the training dataset.

**Table 3.** Five-fold cross-validation test results of anti-CRISPRs prediction.

Metrics	Accuracy	Precision	Recall	F1 score	MCC
AcRanker	0.8752	0.8591	0.8953	0.8666	0.7509
PaCRISPR	0.8087	0.7344	**0.9738**	0.7588	0.6552
DeepAcr	0.7269	0.9447	0.6518	0.7710	0.5093
AcrNET (Transformer only)	0.9326	0.9351	0.9248	0.9299	0.8652
**AcrNET**	**0.9442**	**0.9471**	0.9409	**0.9418**	**0.8883**

AcrNET achieves the best results, outperforming the other methods significantly. AcrNET model with only Transformer features also has relatively good performance, better than PaCRISPR, AcRanker, and DeepAcr. Results in this table are averaged over 10 different random seeds in our experiments, and variances are ˂0.001.

The bold values show the best performance among all the compared methods.

**Table 4.** Cross-dataset test results of anti-CRISPRs prediction with separation 1.

Metrics	TN	FN	FP	TP	Specificity	Accuracy	Precision	Recall	F1 score	MCC
AcRanker	244	157	16	53	0.9385	0.7681	0.2524	0.6319	0.3799	0.2681
PaCRISPR	217	67	43	143	0.8346	0.7660	0.7688	**0.6810**	0.7222	0.5242
DeepAcr	174	73	86	137	0.6692	0.6617	0.6143	0.6524	0.6328	0.3202
AcrNET (Transformer only)	255	110	5	100	**0.9808**	**0.9524**	0.4762	0.7553	0.6349	0.5454
**AcrNET**	232	57	28	143	0.8923	0.7979	**0.8363**	**0.6810**	**0.7505**	**0.5924**

AcrNET accomplishes great improvement compared with the other state-of-the-art computational methods, especially on precision, F1 score, and MCC. The Transformer features contribute greatly to the AcrNET generalization capability, but the other features are also very helpful. Results in this table are averaged over 10 different random seeds in our experiments, and variances are ˂0.001.

The bold values show the best performance among all the compared methods.

Cross-dataset test evaluates the model’s generalization capacity when dealing with testing data with low sequence similarity from training data. From the results of the cross-dataset test, we can observe that both AcRanker and DeepAcr achieve small Precision and F1-score scores, which may be because they tend to predict new sequences as positive and cause wrong predictions. We suppose that positive and negative sequences share some features, whereas the negative sequences do not have the universal features since they are of different types of proteins. It is impossible to learn common features from negative sequences. However, our model can effectively avoid the wrong predictions because the rich knowledge about the extensive database contained in the Transformer learner can help AcrNET make better predictions based on global considerations.

We also compare AcrNET with Gussow et al. (2020). The dataset used for the evaluation was collected from the study of Gussow et al. (2020). There are 3654 Acrs and 4500 non-Acrs in the dataset, identified by unique identifiers. To generate the sequences of these proteins, we searched the NCBI protein database with these identifiers using Entrez (https://www.ncbi.nlm.nih.gov/sites/batchentrez). We found that some identifiers are defined manually and cannot be found by the program. Finally, we obtained 488 positive sequences and sampled 488 balanced negative sequences. We compared the AcRanker, PaCRISPR, DeepAcr, Gussow et al. (2020), and AcrNET with the same dataset. The results are listed in Table 5. We can see AcrNET outperforms all the other methods across different metrics. The result is consistent with the result in 5-fold cross-validation test in Table 3 even the dataset is much smaller, e.g. DeepAcr has good precision value, and AcrNET has the best other metrics. This result further proves the performance of each method on our dataset.

**Table 5.** Five-fold cross-validation test results of Gussow et al. (2020) and AcrNET.

Metrics	Accuracy	Precision	Recall	F1 score	MCC
AcRanker	0.9286	0.9238	0.9324	0.9276	0.8572
PaCRISPR	0.9367	0.9847	0.8920	0.9356	0.8779
DeepAcr	0.6796	**0.9867**	0.6122	0.7553	0.4521
Gussow et al. (2020)	0.8493	0.8913	0.8186	0.8531	0.6992
**AcrNET**	**0.9543**	0.9762	**0.9360**	**0.9553**	**0.9101**

We collected training and testing data from Gussow et al. (2020), used identifiers to fetch sequence data. We finally obtained 488 positive sequences and sampled balanced 488 negative sequences. We compare the previous works and AcrNET with the dataset from Gussow et al. (2020).

The bold values show the best performance among all the compared methods.

### 2.7 Prediction performance on datasets with different similarity

About 95% similarity threshold of training dataset might be too high in practical experiments. We picked samples with 40% and 70% similarity from the positive dataset, which left 238 positive samples with the 40% threshold and 657 samples with the 70% threshold. The similarity among the negative samples was already below 40%. In the 5-fold test, we randomly picked 20% samples for testing. And in cross-dataset test, the testing data from the original dataset remained the testing data, which contained 85 samples in the 40% threshold and 160 samples in the 70% threshold in separation 1, 88 samples in the 40% threshold and 156 samples in 70% threshold in separation 2, and 69 samples in 40% threshold and 108 samples in the 70% threshold in separation 3. We randomly sampled the same size of the positive dataset from the negative dataset for training and testing in 40% and 70% threshold experiments correspondingly. With the same dataset, we compared the performance between AcRanker, PaCRISPR, DeepAcr, and AcrNET. The results are listed in Supplementary Appendix Section 5. As we can see from the tables, AcrNET outperforms AcRanker, PaCRISPR, and DeepAcr, especially in cross-data experiments. Analyzing the TP and TN, we can conclude that AcrNET is relatively table on TP and outperforms on TN. The performance is consistent with 95% similarity dataset experiments. We can see AcRanker performs similarly well on TN and TP, while the model and features are not powerful enough to capture more characteristics of Ars. PaCRISPR is relatively robust on TP and TN, whereas not as satisfying as ActNET, since AcrNET considers more features than PaCRISPR. And DeepAcr can be further improved on TN, we reckon that the features that DeepAcr includes may vary on non-Acr proteins, which makes the model confused to extract common features, which is the same reason as in the previous cross-dataset test.

### 2.8 Evaluation of feature influence on prediction results

We perform ablation studies on different features with the 5-fold cross-validation and cross-dataset tests with the full dataset to evaluate their influence on prediction results, as shown in Fig. 3A and B. We can draw some conclusions from this study. First, the performances of the combination of features are always better than the performances of features alone, which means the three kinds of features complement each other. They all reflect the different aspects of the Acr properties. Second, Transformer features play critical roles in both 5-fold cross-validation and cross-dataset tests. Especially, Transformer features always lead to the best results among all the features and meanwhile can improve the results more significantly than other features. This indicates that the Transformer learning module can effectively learn valuable knowledge from the large database to promote the Acr prediction. Third, due to the task’s difficulty, the structure information prediction would not be 100% accurate, which may influence the final prediction performance and thus should be taken into consideration when using it for prediction. Relatively, evolutionary features and Transformer features are more reliable, which can always promote the downstream tasks, such as Acr prediction. Lastly, using the three features, AcrNET (as given in Fig. 3A and B) is usually better than the shallow learning models (as provided in Tables 3 and 4), which suggests the usefulness of the three features and the effectiveness of deep learning methods for this problem.

Besides, we also conducted comparison experiments between CNN, RNN, and LSTM. We used RNN on varying lengths features, i.e. secondary structure, solvent accessibility and sequence. Then we concatenated the results from RNN with evolutionary features and Transformer features to FCN. We used the same structure in LSTM experiment as RNN. We applied two layers RNN and LSTM with 64 hidden states. Table 6 shows the results of 5-fold cross-validation test. From the result CNN is slightly better than RNN, whereas LSTM might be too sophisticated for these well-defined features. Since LSTM and RNN may forget early features, whereas CNN does not forget and can also capture order information of protein domains in later hidden layers, which enable CNN better performance on Acr prediction problem.

**Table 6.** Model influence on the prediction results.

Metrics	Accuracy	Precision	Recall	F1 score	MCC
RNN + FCN	0.9402	0.9361	0.9423	0.9386	0.8811
LSTM + FCN	0.9220	0.9058	0.9372	0.9178	0.8491
**FCN**	**0.9442**	**0.9471**	**0.9409**	**0.9418**	**0.8883**

We compare the performance between RNN, LSTM, and CNN. We used RNN and LSTM along the features with varying lengths, which are the same features that we used CNN on. And then injected the results from RNN or LSTM to FCN.

The bold values show the best performance among all the compared methods.

### 2.9 Classification performance on Acr classes

To further estimate the capacity of our model in predicting the specific class that an Acr protein belongs to, we utilize the same features in the classification task as described in Section 2.2. The comparison of classification problem on Acr categories and corresponding ablation studies are demonstrated in Supplementary Appendix Section 6, Table 12 and Fig. 3D, respectively. We can observe from the histograms in Fig. 3D that the prediction results obtained using each type of feature are similar, which indicates that the Acr classification task is relatively straightforward when using these inputs. The evolutionary features obtained from PSSM can achieve slightly better results than other features in both single feature and combination with others. The graph may reveal some correlations between Acr classes and their features. To compare AcrNET with the baseline, we adopt the one-versus-rest strategy to AcRanker and PaCRISPR to convert the binary prediction methods to multi-class prediction methods. Because Acrs belonging to the same class are more likely to form motif sequences, Hidden Markov Model (HMM) may also be able to capture these motif features. Thus, we apply HMM on protein sequences and use it as a baseline for Acr classification comparison. AcrNET outperforms these methods across all the metrics, especially on macro-average metrics (improved by around 20% regarding F1 score), suggesting that AcrNET is an unbiased predictor, performing well on the rare Acr classes. Supplementary Appendix Table 13 lists details of all classes. AcrNET has accurate performance on class II-A, I-F, and I-D since they have enough data. Because others class is formed from eight classes, and III-1, III-B, and I-B has only one sample, classification on others class is challenging. Such accurate, detailed predictions from our model can facilitate biological experiments and have the potential of inspiring more insightful studies on the working mechanisms of Acr proteins. In addition to separated predicting whether a protein is an Acr and Acr type classification problems, we also examined non-Acr samples as the sixth class, and keep the five classes in positive Acrs, which means we train the model for predication and classification together. The result in Supplementary Appendix Table 14 shows that separating prediction and classification have better results. If we separate Acr classes, the model needs to learn individual features of each class instead of the common features from Acrs. Especially for others classes, it becomes more challenging to separate them from non-Acr class. The performance on other classes is much worse than in the separated test in Supplementary Appendix Table 13.

### 2.10 AcrNET learns Acr motifs implicitly

**Figure 4.**
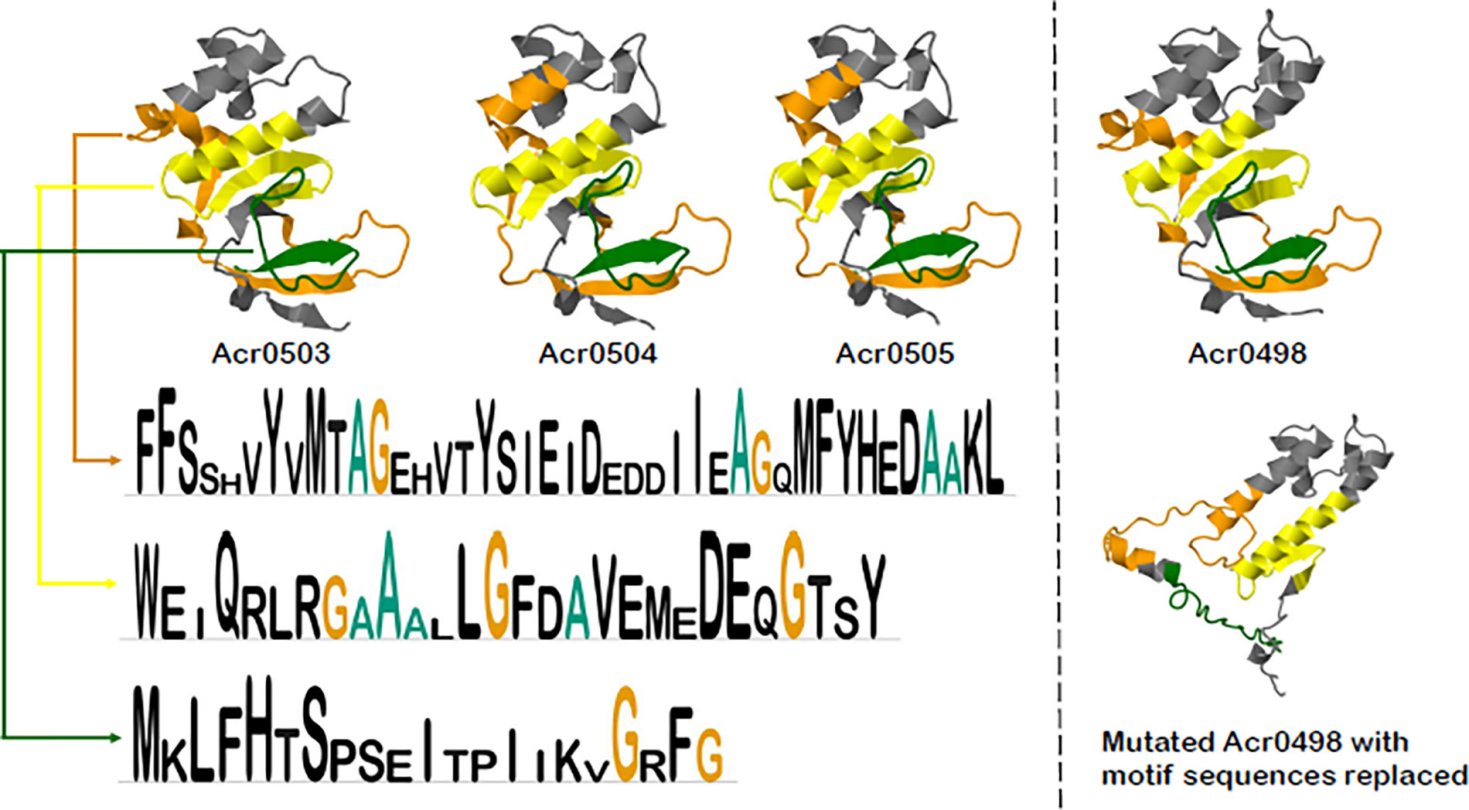
Motif analysis results. The structures of four samples Acr0503, Acr0504, Acr0505, and Acr0498 from the same type AcrFI11 are presented. Three found motifs are listed, and the corresponding structures are colored orange, yellow, and green. The lengths of three motif sequences are 41, 28, and 21. The structure of mutated Acr0498, whose motif sequence is replaced by random sequences, is also shown.

To explain the hidden rule of model prediction, we conduct motif analysis to study the Acr sequence and structure patterns. MEME (Bailey et al. 1994) can find motif sequences in the protein data. Motif analysis tool can be found at https://meme-suite.org/meme/tools/meme. We used MEME under Motif Discovery. Input the protein sequence file and keep other default value to get the results. The results provide overall motif sequences and motif sequences for each protein. Applying MEME to the Acr dataset, we show that Acrs belonging to the same class are more likely to form motif sequences. To further investigate the structure pattern of these motif sequences, we utilize an accurate protein structure prediction method, AlphaFold. Figure 4 presents some motif sequence and structure results. Such structures reveal that these motif sequences correspond to highly similar protein secondary structures, which may serve as the hidden rule for AcrNET. Also, replacing motif sequences will change these important protein secondary structures, which may make positive samples lose Acr-related features. To verify this conjecture, we conduct motif mutation by replacing the motif sequences with random sequences for randomly selected Acrs, including Acr0498 (AcrFI11), Acr0562 (AcrIIA7), Acr0559 (AcrIIA8), and Acr0560 (AcrIIA9). The native Acr sequences and mutated sequences are inputted to AcrNET for Acr prediction. The results show that the native Acrs are all predicted as anti-CRISPR successfully, and all the mutated sequences are predicted as non-anti-CRISPR. Tables 7 and 8 show the confidence scores from the last layer of AcrNET. The above results suggest that AcrNET learns the important motifs of the Acr sequences implicitly, which serves as the foundation of its prediction. Considering rigorousness, we also mutate the non-motif sequences and follow the same steps mentioned above to study their effects. The results from Table 9 show that three mutated sequences are still predicted as anti-CRISPR with only one exception, suggesting that our model indeed captures the important motif information in most Acr sequences.

**Table 7.** AcrNET prediction confidence scores of native Acr sequences.

	Acr0498	Acr0562	Acr0559	Acr0560
Acr	1	0.9881	1	1
Non-Acr	0	0.0119	0	0

**Table 8.** AcrNET prediction confidence scores of mutated Acr sequences with motif sequences replaced by random sequences.

	Acr0498	Acr0562	Acr0559	Acr0560
Acr	0	0.0015	0.0001	0.1105
Non-Acr	1	0.9985	0.9999	0.8895

**Table 9.** AcrNET prediction confidence scores of mutated Acr sequences with non-motif sequences replaced by random sequences.

	Acr0498	Acr0562	Acr0559	Acr0560
Acr	0.0922	1	1	1
Non-Acr	0.9078	0	0	0

### 2.11 Docking methods validate our predictions

**Figure 5.**
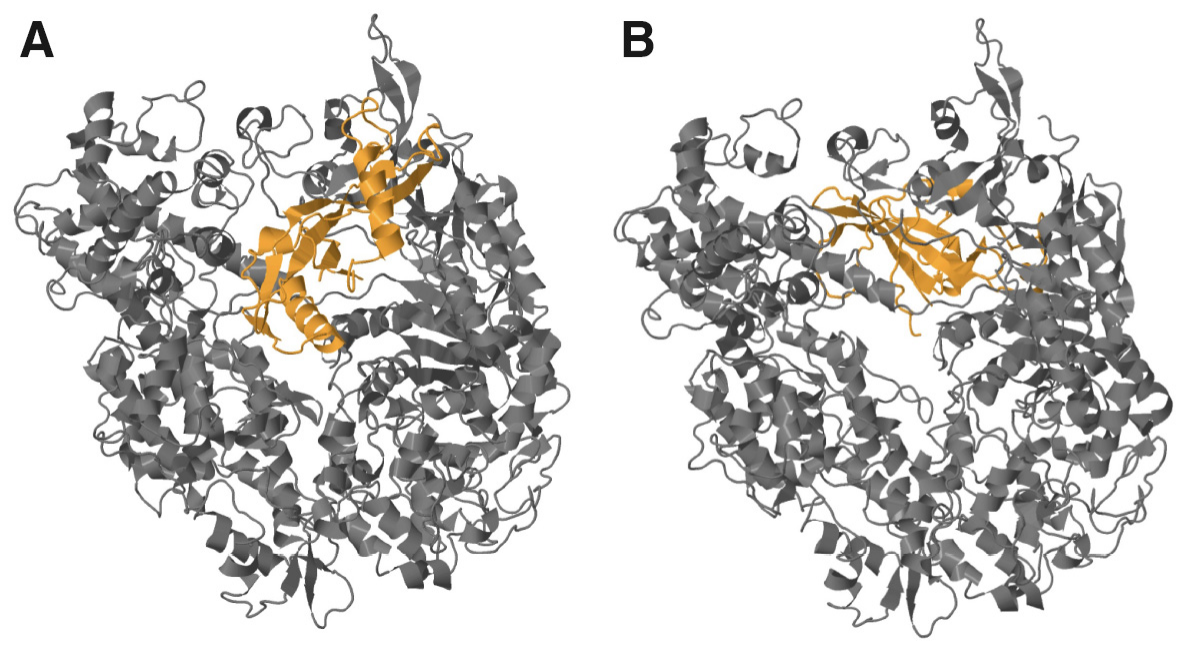
Acr0275 and receptor docking results with HDOCK. We check the docking results of both the native Acr0275(AcrIIA) structure and the mutated one, with the motif sequence replaced and its structure predicted by AlphaFold. The structure in orange is the Acr sample, and the structure in gray is the receptor. (A) Docking result of the native Acr0275 and its receptor. (B) Docking result of the mutated Acr0275 with motif sequences replaced and its receptor.

**Figure 6.**
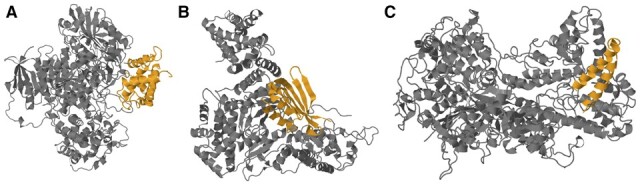
Acr1927, Acr0434, Acr0554, and their receptors docking results with HDOCK. We check the docking results of Acr1927(AcrIF), Acr0434(AcrID), and Acr0554(AcrIIC). The structure in orange is the Acr sample, and the structure in gray is the receptor. (A) Docking result of the Acr1927 and its receptor. (B) Docking result of the Acr0434 and its receptor. (C) Docking result of the Acr0554 and its receptor.

Biological experimental validation is time-consuming and expensive, and we wish to facilitate it with protein–protein docking and perform more comprehensive prediction results. For a new candidate Acr protein, we predict protein structure with AlphaFold and investigate the interaction between the protein and its target using protein–protein docking tools, which could also provide information about the Acr mechanism. We used the official implementation of AlphaFold version 2.0 to perform the protein structure prediction. The package is from: https://github.com/deepmind/alphafold. Following the tutorial, we setup AlphaFold by downloading the genetic databases and model parameters after building the docker environment on our Linux machine. Then AlphaFold can predict the structures for the Acr proteins by taking the sequence FASTA files as input. Here is a slightly simplified version of AlphaFold on Colab Notebook: https://colab.research.google.com/github/deepmind/alphafold/blob/main/notebooks/AlphaFold.ipynb, which is easier to run. As for docking tools, various tools are available including ClusPro (Kozakov et al. 2013, 2017; Vajda et al. 2017; Desta et al. 2020), HDOCK (Huang and Zou 2008, 2014; Yan et al. 2017a, 2017b, 2020), LzerD (Christoffer et al. 2021), and Schrödinger (Zhu et al. 2014). We study the interaction between Acr0275(AcrIIA) from anti-CRISPRdb and its receptor by HDOCK. We mutated the motif sequence from the Acr with random sequences and conducted docking for both the native protein and the mutated one. The docking results are shown in Fig. 5. The docking energy scores increased from −364.51 (predicted as Acr by AcrNET) to −254.79 (predicted as non-Acr by AcrNET), showing the mutated proteins has less stable interaction with the original receptor. Besides, we conducted docking for three more Acrs from different classes, which are Acr1927(AcrIF), Acr0434(AcrID), and Acr0554(AcrIIC). These results are shown in Fig. 6. Their docking energy scores are −403.66, −224.48, and −222.24, respectively, and more negative docking score means a more possible way of Acr–receptor interaction. Those docking results with high negative docking score worth more attention in real biological experimental validation. Understandably, the current docking method may not be 100% accurate, but it could facilitate the discovery and study of Acr proteins, together with AcrNET and AlphaFold. Further improvement on the tools could lead to better toolkits for Acr investigation.

## 3 Conclusion

Performing accurate anti-CRISPR predictions can help us reduce off-target accidents in gene editing and develop phage therapy. Here, we propose a deep learning method, AcrNET, for anti-CRISPR prediction. When receiving a protein sequence as input, AcrNET will accurately estimate whether this sequence is an Acr and further predict its specific type if it is an Acr, assisting biological identification of new Acrs efficiently. Without any prior knowledge, using biological experiments to verify whether a protein sequence is an Acr and its specific category is often very time consuming and expensive, which is nearly impossible with the rapid emergence of a large number of protein sequences. AcrNET overcomes the limitation of the experimental methods that rely heavily on the reaction between CRISPR-Cas and Acr to find new Acrs, thus can find Acrs from the large-scale protein database. Furthermore, the proposed AcrNET can carry out accurate prediction and classification tasks efficiently, which is very important for practical applications in big protein databases. By predicting Acr accurately, AcrNET provides useful prior knowledge for biological researchers to identify Acr more efficiently. To further help the community, we provide an online web server https://proj.cse.cuhk.edu.hk/aihlab/AcrNET/. Due to limited computing power, we only support input files containing no more than three sequences whose length is ˂300. We examined three proteins with a length of 300 and a concurrency setting of 5, the RAM usage reached 49 GB, causing significant performance degradation of our server as it supports other web services as well. Besides, considering only 59 out of 2256 proteins in our dataset exceed 300, we choose 300 as a threshold. For higher computing needs, please download our program from https://github.com/banma12956/AcrNET and run it locally.

AcrNET achieves high accuracy on the Acr prediction tasks. One important reason is that we adopt the Transformer learning algorithm to deal with the problem of data scarcity. Previous methods only study small sizes of Acr datasets, which makes it difficult to learn general and informative patterns. As a result, these models cannot perform accurate Acr predictions and provide limited prior knowledge on protein sequences, which still need a large number of biological experiments for verification, causing an insufficient driving force for practical usage. By introducing the Transformer module, we can successfully generate effective representations by considering the structural information of protein sequences themselves and using the knowledge of the common properties of protein sequences learned from extensive databases in the pre-training process. These informative representations promote the training of the model effectively and improve the final prediction results, which will greatly decrease the time and cost of biological verification. Such an idea and features can also be applied to similar computational problems with limited annotated data.

The proposed AcrNET provides the first computational solution for predicting Acr classes, which has not been studied before. Unlike other methods, which rely on the CRISPR-Cas system to perform predictions, the proposed AcrNET can directly predict the specific types of Acr without such limitation. Experimental results demonstrate that the four evolutionary features result in better prediction performance than other features, indicating that the specific Acr types are more closely related to the protein evolutionary information.

The motif analysis shows the importance of motif sequences and their corresponding structures to Acrs prediction. Acrs belonging to the same category are more likely to have similar motif sequences, and these motif sequences correspond to highly similar protein secondary structures, which are the unique features of this type of Acrs. Such sequence patterns and structure patterns can be learned by AcrNET implicitly and serve as the prediction factors. Experiment results show that Acrs with motif sequences replaced will be predicted as non-Acrs by AcrNET, which indicates the prediction basis of AcrNET.

The success of AlphaFold and protein–protein docking analysis enhance our analysis pipeline. We can simulate the interaction between Acr and CRISPR-Cas proteins by utilizing docking tools before biological experiments. These tools can provide useful information, including docking position and docking energy, which can assist in designing and implementing biological experiments.

Despite the great improvement of AcrNET over the previous methods, our method could be improved further with an even larger dataset. Also, information representation of the protein structure from AlphaFold could boost our model, although currently, it is still time-consuming to run AlphaFold, and efficient protein 3D structure representation is still under exploration. Finally, with the assistance of AlphaFold and docking tools, we can illustrate the Acr mechanism to some extent. However, they are not within the AcrNET model. In the future, it will be desirable to design a comprehensive deep learning model, which can perform Acr prediction and illustrate its mechanisms simultaneously.

## Supplementary data

Supplementary data are available at *Bioinformatics* online.

## Conflict of interest statement

None declared.

## Supplementary Material

btad259_Supplementary_Data

## References

[btad259-B1] Bailey TL , Elkan C. Fitting a mixture model by expectation maximization to discover motifs in bipolymers. Proc Int Conf Intell Syst Mol Biol 1994;2:28–36. https://pubmed.ncbi.nlm.nih.gov/7584402. 7584402

[btad259-B2] Bairoch A , Apweiler R, Wu CH et al The universal protein resource (uniprot). Nucleic Acids Res 2007;35:D193–7. 10.1093/nar/gkm895. 17142230 PMC1669721

[btad259-B3] Bondy-Denomy J , Pawluk A, Maxwell KL et al Bacteriophage genes that inactivate the CRISPR/CAS bacterial immune system. Nature 2013;493:429–32. 10.1038/nature11723 23242138 PMC4931913

[btad259-B4] Chen S , Tan Q, Li J et al USPNet: unbiased organism-agnostic signal peptide predictor with deep protein language model. *bioRxiv*, 2021: 2021–11. 10.1038/s43588-023-00576-2 38177492

[btad259-B5] Christoffer C , Chen S, Bharadwaj V et al Lzerd webserver for pairwise and multiple protein–protein docking. Nucleic Acids Res 2021;49:W359–65. 10.1093/nar/gkab336 33963854 PMC8262708

[btad259-B6] Desta IT , Porter KA, Xia B et al Performance and its limits in rigid body protein–protein docking. Structure 2020;28:1071–81.e3. 10.1016/j.str.2020.06.006 32649857 PMC7484347

[btad259-B7] Devlin J , Chang MW, Lee K et al Bert: pre-training of deep bidirectional transformers for language understanding. ArXiv abs/1810.04805, 2019.

[btad259-B8] Ding S , Li Y, Shi Z et al A protein structural classes prediction method based on predicted secondary structure and psi-blast profile. Biochimie 2014;97:60–5. 10.1016/j.biochi.2013.09.013 24067326

[btad259-B9] Dong C , Hao GF, Hua HL et al Anti-CRISPRdb: a comprehensive online resource for anti-CRISPR proteins. Nucleic Acids Res 2018;46:D393–8. 10.1093/nar/gkx835 29036676 PMC5753274

[btad259-B10] Dong C , Pu DK, Ma C et al Precise detection of acrs in prokaryotes using only six features. *bioRxiv*, 2020:2020–05.

[btad259-B11] Dong Q , Zhou S, Guan J. A new taxonomy-based protein fold recognition approach based on autocross-covariance transformation. Bioinformatics 2009;25:2655–62. 10.1093/bioinformatics/btp500 19706744

[btad259-B12] Eitzinger S , Asif A, Watters KE et al Machine learning predicts new anti-CRISPR proteins. Nucleic Acids Res 2020;48:4698–708. 10.1093/nar/gkaa219 32286628 PMC7229843

[btad259-B13] Gussow AB , Park AE, Borges AL et al Machine-learning approach expands the repertoire of anti-CRISPR protein families. Nat Commun 2020;11:1–12. 10.1038/s41467-020-17652-0 32728052 PMC7391736

[btad259-B14] Hinton G , Osindero S, Welling M et al Unsupervised discovery of nonlinear structure using contrastive back propagation. Cogn Sci 2006;30:725–31. 10.1207/s15516709cog0000_76 21702832

[btad259-B15] Huang SY , Zou X. An iterative knowledge-based scoring function for protein–protein recognition. Protein Struct Funct Bioinform 2008;72:557–79. 10.1002/prot.21949 18247354

[btad259-B16] Huang SY , Zou X. A knowledge-based scoring function for protein–RNA interactions derived from a statistical mechanics-based iterative method. Nucleic Acids Res 2014;42:e55. 10.1093/nar/gku077 24476917 PMC3985650

[btad259-B17] Hynes AP , Rousseau GM, Lemay ML et al An anti-CRISPR from a virulent streptococcal phage inhibits streptococcus pyogenes cas9. Nat Microbiol 2017;2:1374–80. 10.1038/s41564-017-0004-7 28785032

[btad259-B18] Jedhe GS , Arora PS. Hydrogen bond surrogate helices as minimal mimics of protein α-helices. Method Enzymol 2021;656:1–25. 10.1016/bs.mie.2021.04.007. 34325784

[btad259-B19] Kabsch W , Sander C. Dictionary of protein secondary structure: pattern recognition of hydrogen-bonded and geometrical features. Biopolym Original Res Biomol 1983;22:2577–637. 10.1002/bip.360221211 6667333

[btad259-B20] Källberg M , Wang H, Wang S et al Template-based protein structure modeling using the RaptorX web server. Nat Protoc 2012;7:1511–22. 10.1038/nprot.2012.085 22814390 PMC4730388

[btad259-B21] Koonin EV , Makarova KS, Zhang F. Diversity, classification and evolution of CRISPR-Cas systems. Curr Opin Microbiol 2017;37:67–78. 10.1016/j.mib.2017.05.008 28605718 PMC5776717

[btad259-B22] Kozakov D , Beglov D, Bohnuud T et al How good is automated protein docking? Protein Struct Funct Bioinform 2013;81:2159–66. 10.1002/prot.24403 PMC3934018 23996272

[btad259-B23] Kozakov D , Hall DR, Xia B et al The cluspro web server for protein–protein docking. Nat Protoc 2017;12:255–78. 10.1038/nprot.2016.169 28079879 PMC5540229

[btad259-B24] Li Y , Wang S, Umarov R et al DEEPre: sequence-based enzyme EC number prediction by deep learning. Bioinformatics 2018;34:760–9. 10.1093/bioinformatics/btx680 29069344 PMC6030869

[btad259-B25] Liu T , Zheng X, Wang J. Prediction of protein structural class for low-similarity sequences using support vector machine and psi-blast profile. Biochimie 2010;92:1330–4. 10.1016/j.biochi.2010.06.013 20600567

[btad259-B26] Marino ND , Pinilla-Redondo R, Csörgő B et al Anti-CRISPR protein applications: natural brakes for CRISPR-Cas technologies. Nat Methods 2020;17:471–9. 10.1038/s41592-020-0771-6 32203383 PMC8510557

[btad259-B27] Pauling L , Corey RB, Branson HR. The structure of proteins: two hydrogen-bonded helical configurations of the polypeptide chain. Proc Natl Acad Sci USA 1951;37:205–11. 10.1073/pnas.37.4.205 14816373 PMC1063337

[btad259-B28] Pawluk A , Staals RH, Taylor C et al Inactivation of CRISPR-Cas systems by anti-CRISPR proteins in diverse bacterial species. Nat Microbiol 2016;1:1–6. 10.1038/nmicrobiol.2016.85 27573108

[btad259-B29] Pawluk A , Davidson AR, Maxwell KL. Anti-CRISPR: discovery, mechanism and function. Nat Rev Microbiol 2018;16:12–7. 10.1038/nrmicro.2017.120 29062071

[btad259-B30] Radford A , Wu J, Child R et al Language models are unsupervised multitask learners. OpenAI blog 2019;1(8):9.

[btad259-B31] Rao R , Meier J, Sercu T et al Transformer protein language models are unsupervised structure learners. *bioRxiv*, 2021:2020–12.

[btad259-B32] Rauch BJ , Silvis MR, Hultquist JF et al Inhibition of CRISPR-Cas9 with bacteriophage proteins. Cell 2017;168:150–8.e10. 10.1016/j.cell.2016.12.009 28041849 PMC5235966

[btad259-B33] Rives A , Meier J, Sercu T et al Biological structure and function emerge from scaling unsupervised learning to 250 million protein sequences. Proc Natl Acad Sci USA 2021;118:e2016239118. 10.1073/pnas.2016239118 33876751 PMC8053943

[btad259-B34] Sledzieski S , Singh R, Cowen L et al Sequence-based prediction of protein–protein interactions: a structure-aware interpretable deep learning model. bioRxiv, 2021:2021–01. 10.1016/j.cels.2021.08.010 PMC8586911 34536380

[btad259-B35] Stanley SY , Maxwell KL. Phage-encoded anti-crispr defenses. Annu Rev Genet 2018;52:445–64. 10.1146/annurev-genet-120417-031321 30208287

[btad259-B36] Suzek BE , Wang Y, Huang H et al; UniProt Consortium. Uniref clusters: a comprehensive and scalable alternative for improving sequence similarity searches. Bioinformatics 2015;31:926–32. 10.1093/bioinformatics/btu739 25398609 PMC4375400

[btad259-B37] Vajda S , Yueh C, Beglov D et al New additions to the clusPro server motivated by CAPRI. Protein Struct Funct Bioinform 2017;85:435–44. 10.1002/prot.25219 PMC5313348 27936493

[btad259-B38] Wandera KG , Alkhnbashi OS, Bassett H V et al Anti-CRISPR prediction using deep learning reveals an inhibitor of cas13b nucleases. Mol Cell 2022;82:2714–26.e4. 10.1016/j.molcel.2022.05.003 35649413

[btad259-B39] Wang J , Yang B, Revote J et al POSSUM: a bioinformatics toolkit for generating numerical sequence feature descriptors based on PSSM profiles. Bioinformatics 2017;33:2756–8. 10.1093/bioinformatics/btx302 28903538

[btad259-B40] Wang J , Dai W, Li J et al PaCRISPR: a server for predicting and visualizing anti-CRISPR proteins. Nucleic Acids Res 2020;48:W348–57. 10.1093/nar/gkaa432 32459325 PMC7319593

[btad259-B41] Wang J , Dai W, Li J et al AcrHub: an integrative hub for investigating, predicting and mapping anti-CRISPR proteins. Nucleic Acids Res 2021;49:D630–8. 10.1093/nar/gkaa951 33137193 PMC7779044

[btad259-B42] Yan Y , Wen Z, Wang X et al Addressing recent docking challenges: a hybrid strategy to integrate template-based and free protein–protein docking. Protein Struct Funct Bioinform2017a;85:497–512. 10.1002/prot.25234 28026062

[btad259-B43] Yan Y , Zhang D, Zhou P et al HDOCK: a web server for protein–protein and protein–DNA/RNA docking based on a hybrid strategy. Nucleic Acids Res2017b;45:W365–73. 10.1093/nar/gkx407 28521030 PMC5793843

[btad259-B44] Yan Y , Tao H, He J et al The HDOCK server for integrated protein–protein docking. Nat Protoc 2020;15:1829–52. 10.1038/s41596-020-0312-x 32269383

[btad259-B45] Yi H , Huang L, Yang B et al AcrFinder: genome mining anti-CRISPR operons in prokaryotes and their viruses. Nucleic Acids Res 2020;48:W358–65. 10.1093/nar/gkaa351 32402073 PMC7319584

[btad259-B46] Yu Q , Dong Z, Fan X et al HMD-AMP: Protein language-powered hierarchical multi-label deep forest for annotating antimicrobial peptides. arXiv:2111.06023, arXiv preprint, 2021.

[btad259-B47] Zhu K , Day T, Warshaviak D et al Antibody structure determination using a combination of homology modeling, energy-based refinement, and loop prediction. Protein Struct Funct Bioinform 2014;82:1646–55. 10.1002/prot.24551 PMC5282925 24619874

[btad259-B48] Zou L , Nan C, Hu F. Accurate prediction of bacterial type IV secreted effectors using amino acid composition and PSSM profiles. Bioinformatics 2013;29:3135–42. 10.1093/bioinformatics/btt554 24064423 PMC5994942

[btad259-B49] Zou Z , Tian S, Gao X et al MlDEEPre: multi-functional enzyme function prediction with hierarchical multi-label deep learning. Front Genet 2018;9:714. 10.3389/fgene.2018.00714 30723495 PMC6349967

