# Supplementary material for: AcrNET: predicting anti-CRISPR with deep learning
Source: Bioinformatics. 2023 Apr 21;39(5):btad259. doi: 10.1093/bioinformatics/btad259 (PMC10174705; doi:10.1093/bioinformatics/btad259)
Supplement: btad259_Supplementary_Data [file btad259_supplementary_data.pdf]

Appendix

1 INPUT DATA ORGANIZATION

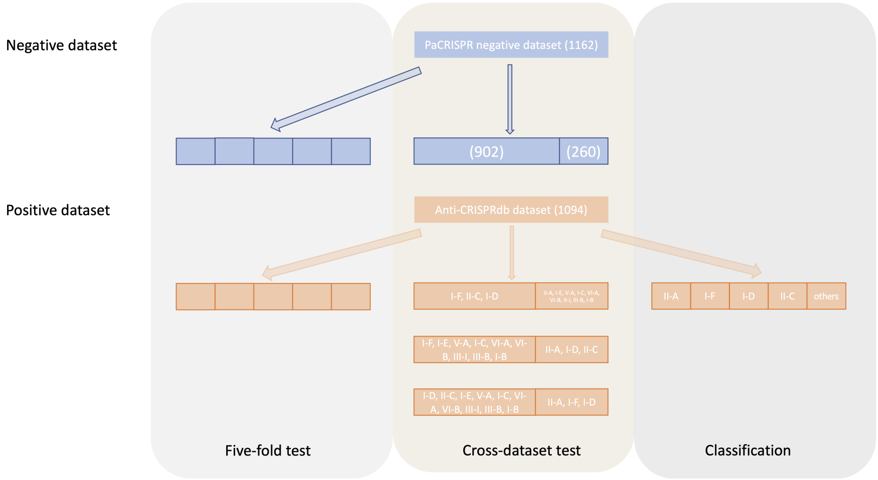

Fig. 1. **The organization of our dataset.** We gather dataset from PaCIRSPR and Anti-CIRSPRdb and separate according to five-fold test, cross-dataset test and classification problem.

2 APPLICATION NOTE OF ACRNET

Features. Here we provide more details about our features.

Protein sequence. We utilize the one-hot encoding technique to represent a protein sequence as an $L \times 20$ matrix, where L indicates the length of the sequence and 20 is the number of native amino acid types.

Evolutionary features. We utilize the POSSUM toolkit [10] to obtain the PSSM evolutionary features, which first calculates the PSSM matrix using the PSI-BLAST program via multiple iterations with a certain E-value [9] and then extracts the four features, namely PSSM-composition, DPC-PSSM, PSSM-AC, and RPSSM. In our implementation, we use the default setting, including the UniRef50 database, and the iteration number and E-value are set as 3 and 0.001, respectively.

Secondary structure features. We extract two types of secondary structure features to consider the local folding information of protein fully. We first consider the traditional secondary structure that can be divided into three classes, namely two regular types alpha-helix (H), beta-strand (E), and one irregular type coil region (C) [6]. Then, we further consider the extended secondary structure with eight classes, namely 3_{10} helix (G), alpha-helix (H), pi-helix (I), beta-strand (E), beta-bridge (B), beta-turn (T), high curvature loop (S), and irregular (L) [2]. These two types of secondary structure features are transformed into matrices with shapes of $L \times 3$ and $L \times 8$ respectively using one-hot encoding techniques, which are then combined to consider more secondary structure information. In our implementation, we adopt the RaptorX tool [3] to predict secondary structure.

Author's address:

Solvent accessibility. Three states of solvent accessibility are derived from two thresholds, namely buried (0-10%), medium (11%-40%), and exposed (41%-100%). These features are then encoded into an $L \times 3$ matrix and appended to the matrices of the secondary structure mentioned in the previous part. Similarly, the RaptorX tool in Källberg et al. [3] is utilized to calculate the solvent accessibility information.

Transformer feature. Finally, we utilize the ESM-1b Transformer to calculate Transformer features, which trained a 33-layer Transformer model on the UR50/S dataset with 250 million sequences by comparing the results of Transformer models with different sizes and training datasets pretrained by the authors of [7, 8]. This Transformer module consists of 33 encoder blocks, each of which contains a multi-headed self-attention unit and a feed-forward network unit. Specifically, the objective function is designed as follows to minimize the masked language modeling (MLM) loss:

$$\mathcal{L}_{MLM} = \mathbb{E}_{x \sim X} \mathbb{E}_{M} \sum_{i \in M} \log p(x_i | x_M), \tag{1}$$

where x represents a protein sequence; M is the set of mask indices; x_i denotes a protein sequence with mask token at index i. In our implementation, the outputs of the last encoder block are used. To deal with the issue of unequal protein sequence lengths, we calculate the mean values of the hidden states of all tokens and recorded them as the Transformer features.

***Implementation details of AcrNET.*.** Here we list the details of our model.

Hardware. The proposed AcrNET, which mainly consists of CNN and FCN, is implemented with Python 3.7 and PyTorch 1.8 [5], and is trained on NVIDIA GeForce RTX 3090.

Architecture details. The one-hot encoded inputs, such as sequence, secondary structure, and relative accessibility, are concatenated together, then further processed by a 2D CNN to learn more high-level and informative features. For convenience, the kernel width of the CNN module is set the same as that of the concatenated feature. Max-pooling is connected after the CNN layer. The four evolutionary features and Transformer features were first injected into a 2-layer FCN, then concatenated with the high-level features learned from the one-hot encoded features via CNN. The concatenated features are finally inputted into another FCN layer, which has two-dimensional outputs for the Acr prediction task while has five-dimensional outputs for the Acr classification task. The detailed architecture is shown in Table 1.

Training. During the training process, the batch size is set as 16, the number of epochs is 3000, and the learning rate is set as 0.001. We picked the number of epochs from 30, 300, 3000, and the learning rate from 0.1, 0.01, 0.001, 0.0001. We utilize the Adam [4] provided by PyTorch as the optimizer to train the model. 3000 epochs complete in about 179.22 seconds and inference time is 0.013 seconds. A total of 2256 sequences require 202.62 seconds to compute Transformer features. ESM-1b was trained for 56 epochs, 8.5 hours on 64 GPUs for each epoch. To deal with the imbalance issue in the Acr classification tasks, we select each sequence with different weights to ensure each class has the same probability of being sampled.

Implementation details of baselines. Here we provide the implementation of the methods that we compare with.

AcRanker. The author provides the code on https://github.com/amina01/AcRanker. We simply adopt the implementation from their code.

PaCRISPR. Thanks to the author of PaCRISPR, they provided their source code for 5-fold cross-validation and cross-dataset tests and patient guidance.

Table 1. The detailed architecture of AcrNET in prediction and classification problems.

Layer Type	Size
convolution	channel = 20, kernel size = 5*34, stride = (1, 1)
Max Pooling	
ReLU	
Linear	2390 * 256
Relu	
Linear	256 * 32
Linear	52 * 128
ReLU	
Linear	Prediction: 128 * 2. Classification: 128 * 5

DeepAcr. The authors of DeepAcr provided source code on https://github.com/BackofenLab/DeepAcr. With their model architectures of LSTM, Linear and GRU and the same combination of the models in their evaluation code, we used Adam from PyTorch as the optimizer with the learning rate 0.001, batch size 30 (same as the source code) and epoch 75. We trained each model with our data and used the mean value of the models for evaluation.

Gussow et al. Since the features in the paper are obtained in biological experiments, which is beyond our ability, we only use the dataset from their paper, and apply five-fold cross-validation test. The authors provided well-implemented source code on https://github.com/gussow/acr.

3 CROSS-DATASET TEST WITH SEPARATION 2 AND 3

Table 2. **Cross-dataset test results of anti-CRISPRs prediction with separation 2.**

Metrics	TN	FN	FP	TP	Specificity	Accuracy	Precision	Recall	F1 score	MCC
AcRanker	243	137	17	53	0.9385	0.7571	0.2789	0.6578	0.4077	0.2910
PaCRISPR	227	59	33	131	0.8731	0.7988	0.6895	**0.7956**	0.7401	**0.5773**
DeepAcr	153	57	107	133	0.5885	0.6356	0.5542	0.7000	0.6186	0.2856
AcrNET (Transformer only)	249	97	11	93	**0.9577**	**0.8942**	0.4895	0.7600	0.6327	0.5239
**AcrNET**	217	63	43	127	0.8346	0.7644	**0.7471**	0.6684	**0.7056**	0.5125

Table 3. **Cross-dataset test results of anti-CRISPRs prediction with separation 3.**

Metrics	TN	FN	FP	TP	Specificity	Accuracy	Precision	Recall	F1 score	MCC
AcRanker	238	109	22	23	0.9154	0.5111	0.1742	0.6658	0.2599	0.1329
PaCRISPR	224	32	36	100	0.8615	0.7353	**0.7576**	0.8265	**0.7463**	**0.6147**
DeepAcr	166	68	94	64	0.6385	0.5867	0.4051	0.4848	0.4414	0.1188
AcrNET (Transformer only)	248	91	12	41	**0.9538**	**0.7736**	0.3106	0.7372	0.4432	0.3655
**AcrNET**	162	8	98	124	0.6231	0.7296	0.5586	**0.9394**	0.7006	0.5364

4 MOTIF RESULTS

Motif. Here we provide more motif results. We selected sub-classes with sample size greater than or equal to 20 for motif finding. Finally, six sub-classes are selected. They are AcrIIA7(499 samples), AcrIIA8(48 samples), AcrIIA9(194 samples), AcrIIA11(20 samples), AcrIF11(68 samples) and AcrID(46 samples). All the motifs are found by MEME[1] with default setting, and according to the setting, three motifs are found for each class. The results are shown in Fig.2-7.

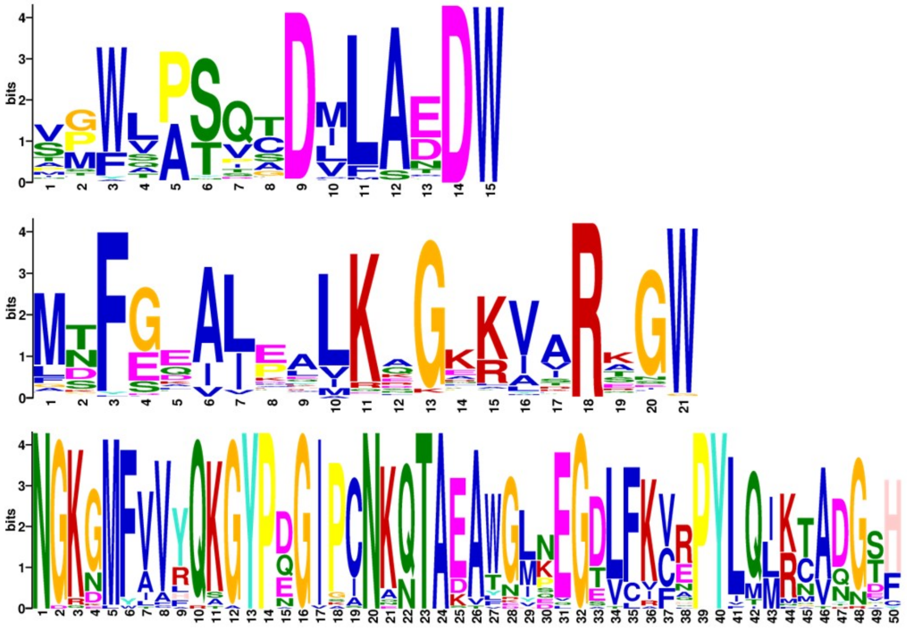

Fig. 2. **Motif results of AcrIIA7**

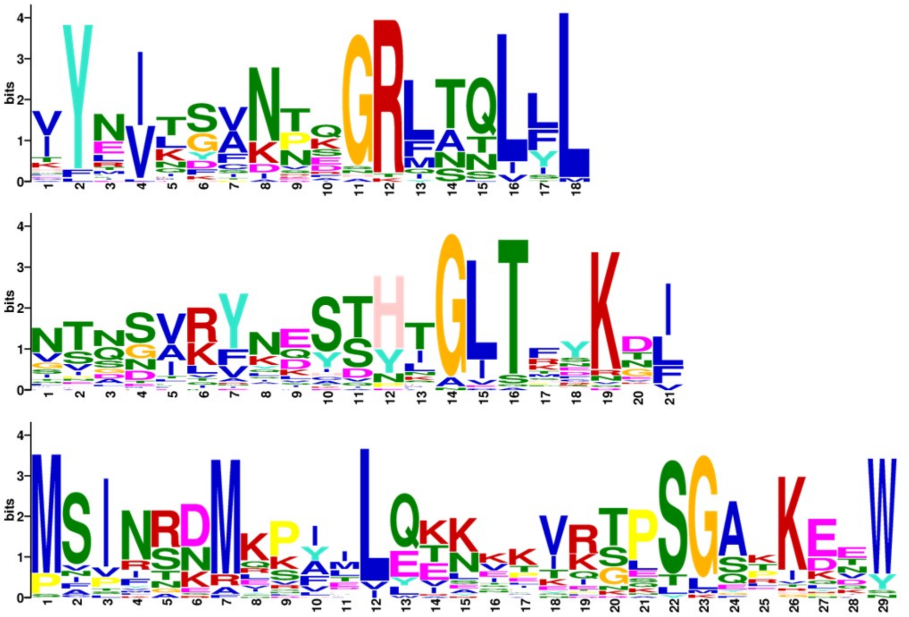

Fig. 3. **Motif results of AcrIIA8**

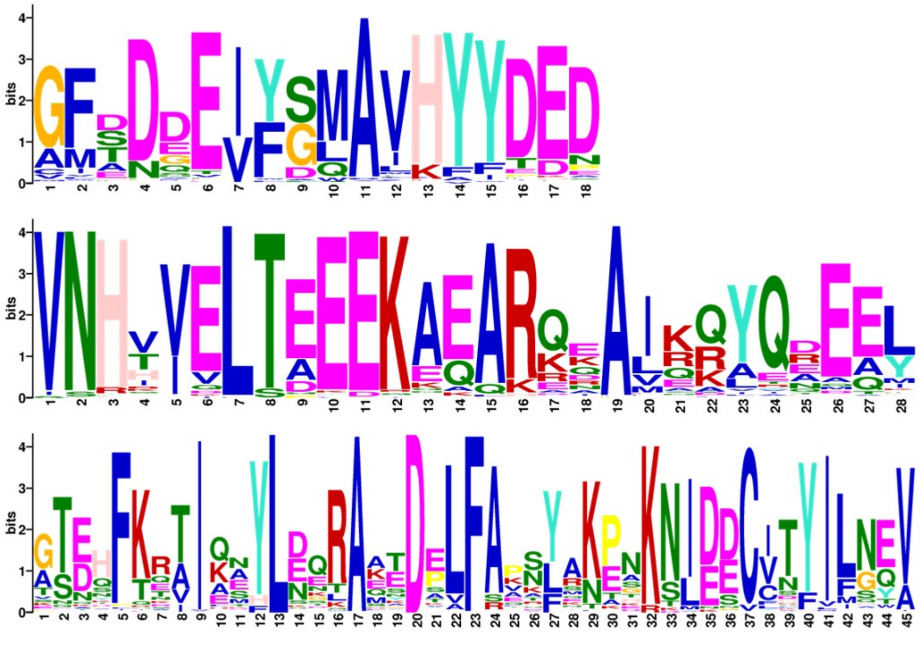

Fig. 4. **Motif results of AcrIIA9**

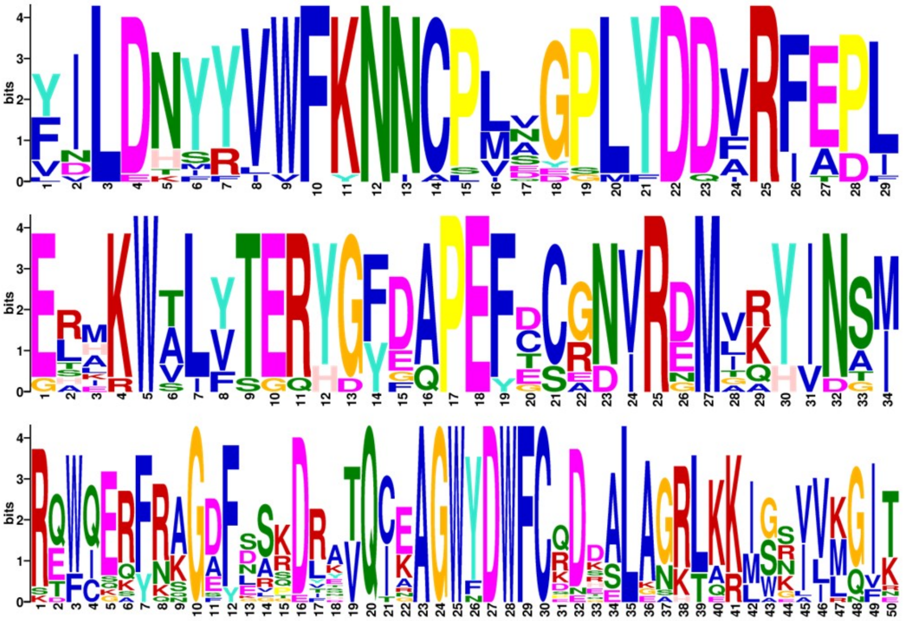

Fig. 5. **Motif results of AcrIIA11**

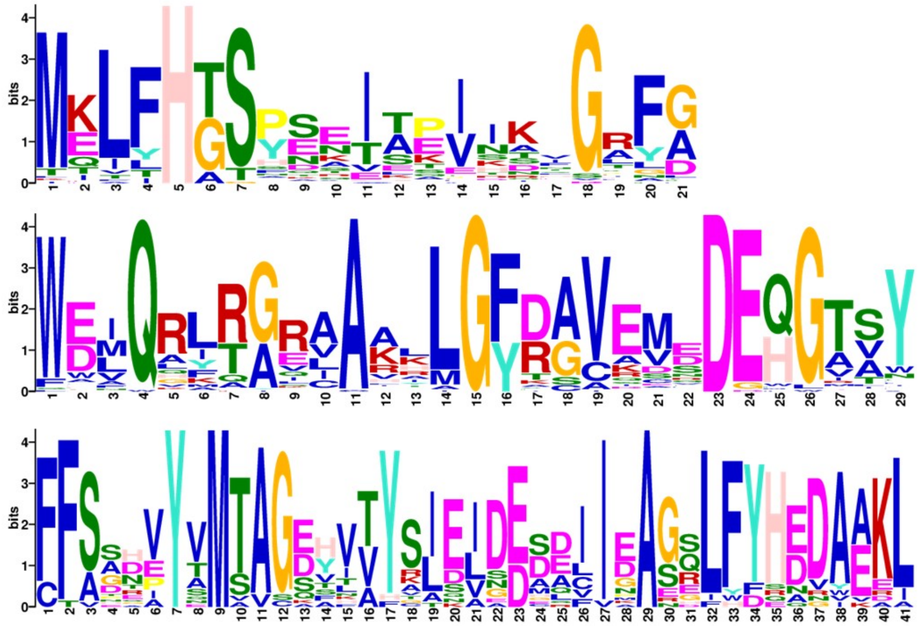

Fig. 6. **Motif results of AcrIF11**

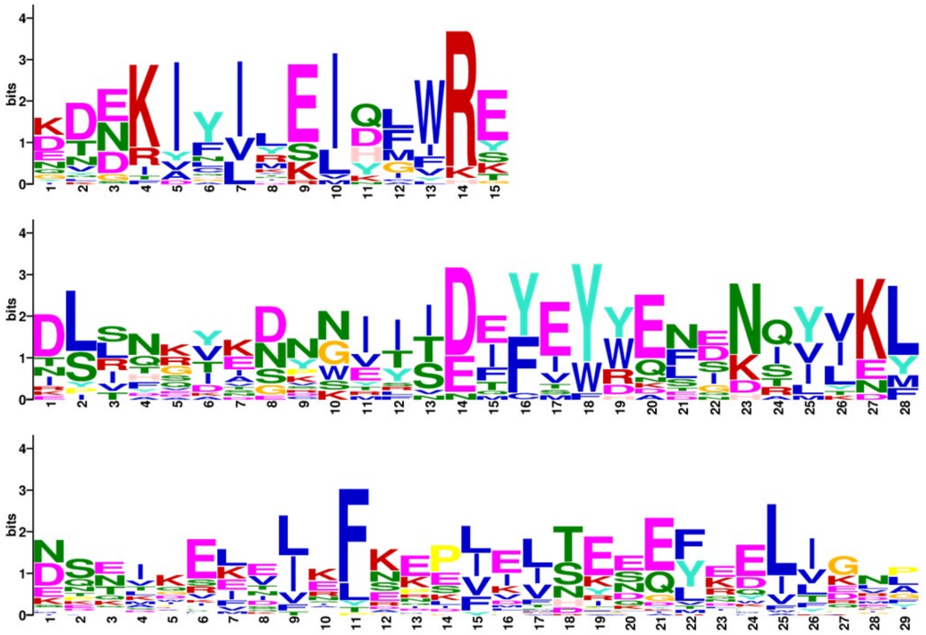

Fig. 7. **Motif results of AcrID1**

5 FIVE-FOLD CROSS-VALIDATION TEST

Table 4. **Five-fold cross-validation test results with 40% similarity dataset.** We used dataset with 40% and 70% similarity and compared the performance. We used the same training and testing dataset split as previous experiments. AcrNET also outperforms other methods, the results are consistent with the previous performance.

Metrics	Accuracy	Precision	Recall	F1 score	MCC
AcRanker	0.7050	0.6837	0.7272	0.7040	0.4120
PaCRISPR	0.8369	**0.9106**	0.7829	0.8393	0.6844
DeepAcr	0.6217	0.8666	0.5888	0.6970	0.2824
**AcrNET**	**0.8840**	0.8840	**0.8859**	**0.8845**	**0.7687**

Table 5. Cross-dataset test results with 40% similarity dataset and separation 1.

Metrics	TN	FN	FP	TP	Specificity	Accuracy	Precision	Recall	F1 score	MCC
AcRanker	58	21	27	64	0.6824	0.7033	0.7529	0.7176	0.7273	0.4364
PaCRISPR	66	15	19	70	0.7765	**0.7865**	0.8235	0.8000	**0.8046**	**0.6007**
DeepAcr	24	11	61	74	0.2824	0.5765	0.5481	**0.8706**	0.6727	0.1891
**AcrNET**	77	30	8	55	**0.9059**	0.7765	**0.8730**	0.6471	0.7432	0.5724

Table 6. Cross-dataset test results with 40% similarity dataset and separation 2.

Metrics	TN	FN	FP	TP	Specificity	Accuracy	Precision	Recall	F1 score	MCC
AcRanker	69	35	19	53	0.7841	0.7361	0.6023	0.6932	0.6625	0.3929
PaCRISPR	69	33	19	55	0.7841	0.7432	0.6250	0.7045	0.6790	0.4144
DeepAcr	42	27	46	61	0.4772	0.5852	0.5701	0.6932	0.6256	0.1746
**AcrNET**	76	17	12	71	**0.8636**	**0.8352**	**0.8554**	**0.8068**	**0.8304**	**0.6715**

Table 7. Cross-dataset test results with 40% similarity dataset and separation 3.

Metrics	TN	FN	FP	TP	Specificity	Accuracy	Precision	Recall	F1 score	MCC
AcRanker	47	37	22	32	0.6811	0.5926	0.4638	0.5725	0.5203	0.1485
PaCRISPR	56	19	13	50	0.8116	0.7937	0.7246	0.7681	0.7576	0.5383
DeepAcr	18	12	51	57	0.2609	0.5435	0.5278	0.8261	0.6441	0.1054
**AcrNET**	64	9	5	60	**0.9275**	**0.8986**	**0.9231**	**0.8696**	**0.8955**	**0.7984**

Table 8. Five-fold cross-validation test results with 70% similarity dataset.

Metrics	Accuracy	Precision	Recall	F1 score	MCC
AcRanker	0.8560	0.8228	0.8730	0.8466	0.7126
PaCRISPR	0.8799	**0.9788**	0.8703	0.9212	0.6933
DeepAcr	0.6211	0.9171	0.5798	0.7090	0.2965
**AcrNET**	**0.9280**	0.9480	**0.9117**	**0.9292**	**0.8572**

Table 9. Cross-dataset test results with 70% similarity dataset and separation 1.

Metrics	TN	FN	FP	TP	Specificity	Accuracy	Precision	Recall	F1 score	MCC
AcRanker	135	88	25	72	0.8438	**0.7423**	0.4500	0.6469	0.5603	0.3196
PaCRISPR	128	105	32	55	0.8000	0.6322	0.3438	0.5719	0.4453	0.1615
DeepAcr	53	43	107	117	0.3313	0.5312	0.5223	**0.7312**	0.6094	0.0682
**AcrNET**	155	84	5	76	**0.9688**	0.7219	**0.9383**	0.4750	**0.6307**	**0.5103**

Table 10. Cross-dataset test results with 70% similarity dataset and separation 2.

Metrics	TN	FN	FP	TP	Specificity	Accuracy	Precision	Recall	F1 score	MCC
AcRanker	141	103	15	53	0.9038	0.7794	0.3397	0.6218	0.4732	0.2950
PaCRISPR	125	52	31	104	0.8013	0.7704	0.6667	0.7340	0.7148	0.4722
DeepAcr	61	36	95	120	0.3910	0.5801	0.5581	0.7692	0.6469	0.1731
**AcrNET**	148	34	8	122	**0.9487**	**0.8654**	**0.9385**	**0.7821**	**0.8531**	**0.7411**

Table 11. Cross-dataset test results with 70% similarity dataset and separation 3.

Metrics	TN	FN	FP	TP	Specificity	Accuracy	Precision	Recall	F1 score	MCC
AcRanker	96	79	12	29	0.8889	0.7073	0.2685	0.5787	0.3893	0.2007
PaCRISPR	84	31	24	77	0.7778	0.7624	0.7130	**0.7454**	0.7368	0.4918
DeepAcr	53	35	55	73	0.4907	0.5833	0.5703	0.6759	0.6186	0.1696
**AcrNET**	102	36	6	72	**0.9444**	**0.8056**	**0.9231**	0.6667	**0.7742**	**0.6361**

6 DETAILED CLASSIFICATION PERFORMANCE

Table 12. **Detailed class prediction performance comparison.** We adopt the one-vs-rest strategy for AcRanker and PaCRISPR, converting binary classification methods to five-class classification methods, and compare their performance on the class prediction problem with AcrNET ("mi":micro-average, "ma": macro-average). AcrNET outperforms the other methods across all the evaluation criteria significantly and consistently, especially on macro-average, suggesting that AcrNET is an unbiased predictor for small classes. Results in this table are averaged over 10 different random seeds in our experiments.

	Accuracy (mi)	Accuracy (ma)	Precision (ma)	Recall (ma)	F1 score (ma)
AcRanker	0.8903	0.6318	0.8532	0.6318	0.6830
PaCRISPR	0.8903	0.5552	0.7071	0.5552	0.5911
HMM	0.8600	0.4994	0.7755	0.4994	0.5607
**AcrNET**	**0.9480**	**0.8583**	**0.8676**	**0.8583**	**0.8531**

Table 13. **Each class prediction performance in AcrNET.** We compare classification performance of each class in AcrNET.

Classes	Accuracy	Precision	Recall	F1 score
II-A	0.9801	0.9875	0.9802	0.9836
I-F	0.9199	0.8414	0.9199	0.8768
I-D	0.9889	0.9567	0.9889	0.9710
II-C	0.7915	0.7892	0.7916	0.7646
others	0.6110	0.7630	0.6110	0.6696

Table 14. **Use non-Acr as the sixth class in classification.** We add non-Acr samples as the sixth class and classify all data together. In this case we can solve prediction and classification problem at the same time. Whereas the performance is not as good as separate prediction and classification.

Classes	Accuracy	Precision	Recall	F1 score
II-A	0.9690	0.9836	0.9690	0.9761
I-F	0.8770	0.7848	0.8770	0.8148
I-D	0.9243	0.8938	0.9243	0.8999
II-C	0.7279	0.8475	0.7279	0.7622
others	0.3386	0.5489	0.3386	0.3932
non-Acr	0.9554	0.9483	0.9554	0.9516
Macro	0.7987	0.8344	0.7987	0.7996
Weighted	0.9372	0.9399	0.9372	0.9356

REFERENCES

[1] Timothy L Bailey, Charles Elkan, et al. 1994. Fitting a mixture model by expectation maximization to discover motifs in bipolymers. (1994).

[2] Wolfgang Kabsch and Christian Sander. 1983. Dictionary of protein secondary structure: pattern recognition of hydrogen-bonded and geometrical features. *Biopolymers: Original Research on Biomolecules* 22, 12 (1983), 2577–2637.

[3] Morten Källberg, Haipeng Wang, Sheng Wang, Jian Peng, Zhiyong Wang, Hui Lu, and Jinbo Xu. 2012. Template-based protein structure modeling using the RaptorX web server. *Nature protocols* 7, 8 (2012), 1511–1522.

[4] Diederik P Kingma and Jimmy Ba. 2014. Adam: A method for stochastic optimization. *arXiv preprint arXiv:1412.6980* (2014).

[5] Adam Paszke, Sam Gross, Francisco Massa, Adam Lerer, James Bradbury, Gregory Chanan, Trevor Killeen, Zeming Lin, Natalia Gimelshein, Luca Antiga, et al. 2019. Pytorch: An imperative style, high-performance deep learning library. *Advances in neural information processing systems* 32 (2019), 8026–8037.

[6] Linus Pauling, Robert B Corey, and Herman R Branson. 1951. The structure of proteins: two hydrogen-bonded helical configurations of the polypeptide chain. *Proceedings of the National Academy of Sciences* 37, 4 (1951), 205–211.

[7] Alexander Rives, Joshua Meier, Tom Sercu, Siddharth Goyal, Zeming Lin, Jason Liu, Demi Guo, Myle Ott, C Lawrence Zitnick, Jerry Ma, et al. 2021. Biological structure and function emerge from scaling unsupervised learning to 250 million protein sequences. *Proceedings of the National Academy of Sciences* 118, 15 (2021).

[8] Baris E Suzek, Hongzhan Huang, Peter McGarvey, Raja Mazumder, and Cathy H Wu. 2007. UniRef: comprehensive and non-redundant UniProt reference clusters. *Bioinformatics* 23, 10 (2007), 1282–1288.

[9] Jiawei Wang, Wei Dai, Jiahui Li, Ruopeng Xie, Rhys A Dunstan, Christopher Stubenrauch, Yanju Zhang, and Trevor Lithgow. 2020. PaCRISPR: a server for predicting and visualizing anti-CRISPR proteins. *Nucleic acids research* 48, W1 (2020), W348–W357.

[10] Jiawei Wang, Bingjiao Yang, Jerico Revote, Andre Leier, Tatiana T Marquez-Lago, Geoffrey Webb, Jiangning Song, Kuo-Chen Chou, and Trevor Lithgow. 2017. POSSUM: a bioinformatics toolkit for generating numerical sequence feature descriptors based on PSSM profiles. *Bioinformatics* 33, 17 (2017), 2756–2758.
